# Modification of Aβ Peptide Aggregation via Covalent Binding of a Series of Ru(III) Complexes

**DOI:** 10.3389/fchem.2019.00838

**Published:** 2019-12-03

**Authors:** Luiza M. F. Gomes, Janaina C. Bataglioli, Allison J. Jussila, Jason R. Smith, Charles J. Walsby, Tim Storr

**Affiliations:** Department of Chemistry, Simon Fraser University, Burnaby, BC, Canada

**Keywords:** dementia, Alzheimer's disease, amyloid-beta peptide, Ru(III) complexes, peptide aggregation

## Abstract

Alzheimer's disease (AD) is the most common form of dementia, leading to loss of cognition, and eventually death. The disease is characterized by the formation of extracellular aggregates of the amyloid-beta (Aβ) peptide and neurofibrillary tangles of tau protein inside cells, and oxidative stress. In this study, we investigate a series of Ru(III) complexes (**Ru-N**) derived from NAMI-A in which the imidazole ligand has been substituted for pyridine derivatives, as potential therapeutics for AD. The ability of the **Ru-N** series to bind to Aβ was evaluated by NMR and ESI-MS, and their influence on the Aβ peptide aggregation process was investigated via electrophoresis gel/western blot, TEM, turbidity, and Bradford assays. The complexes were shown to bind covalently to the Aβ peptide, likely via a His residue. Upon binding, the complexes promote the formation of soluble high molecular weight aggregates, in comparison to peptide precipitation for peptide alone. In addition, TEM analysis supports both amorphous and fibrillar aggregate morphology for **Ru-N** treatments, while only large amorphous aggregates are observed for peptide alone. Overall, our results show that the **Ru-N** complexes modulate Aβ peptide aggregation, however, the change in the size of the pyridine ligand does not substantially alter the Aβ aggregation process.

## Introduction

Dementias are disorders in which severe cognitive impairment occurs (Gaggelli et al., 2006; Crouch and Barnham, 2012; DeToma et al., 2012) affecting over 50 million people worldwide (Budimir, 2011; Crouch and Barnham, 2012; WHO, 2012). An increase in life expectancy is expected to lead to a sharp increase in the number of dementia cases over the next 20 years (Alzheimer's Association, 2019). Alzheimer's disease (AD), the most common type of dementia, represents 60–70% of dementia cases (Martin Prince et al., 2015), resulting in a significant burden to healthcare systems around the globe. AD is a neurodegenerative disease where protein misfolding and aggregation combined with oxidative stress causes neuronal cell death, leading to loss of cognition and eventually death (Crouch and Barnham, 2012; Rodriguez-Rodriguez et al., 2012; Lee et al., 2014). Currently, treatment strategies for most neurodegenerative diseases are very limited, and approved treatments for AD only ameliorate the symptoms at early to moderate stages of the disease, making this an important research area (Roberson and Mucke, 2006; Adlard et al., 2009; Citron, 2010; Finder, 2010; Selkoe, 2011; Hickey and Donnelly, 2012; Soto and Pritzkow, 2018; Savelieff et al., 2019).

The major neuropathological hallmarks of AD are the aggregation of two proteins, amyloid-β (Aβ) and tau, with the first forming aggregates (oligomers and plaques) in the extracellular environment of the brain, and the latter forming neurofibrillary tangles (NFTs) in neurons due to hyperphosphorylation and oxidative modifications of tau (Um et al., 2013). It is still unclear if these hallmarks are a cause or an effect of AD, however post-mortem examination of the brain in AD patients has shown that Aβ-plaques and NFTs are present (Querfurth and LaFerla, 2010). Interestingly, smaller, soluble Aβ oligomers have been more strongly linked to memory loss and progression of the disease in comparison to plaques. These species have been implicated in the initiation of the processes of oxidative stress, decreased cerebral blood flow, neuronal hyperactivity, synapse deterioration, and nerve cell death (McLean et al., 1999; Lesne et al., 2006; Watt et al., 2013; Heffern et al., 2014; Nortley et al., 2019; Zott et al., 2019).

As cofactors in metalloenzymes, metal ions such as Zn, Cu and Fe are central to many processes in healthy organisms. However, their dyshomeostasis has been observed in neurodegenerative diseases, such as AD (Curtain et al., 2001; Sung et al., 2006; Brown, 2009; Kepp, 2012; Savelieff et al., 2013; Hane and Leonenko, 2014; Ward et al., 2015). A high concentration of these metal ions are present in Aβ plaques (Savelieff et al., 2013), where they are found coordinated typically to His^6, 13, or14^ residues, although Asp^1^, Tyr^10^, and Glu^11^ have been shown to be involved in Aβ peptide metal binding (Miller et al., 2010, 2012; Parthasarathy et al., 2011; Hane and Leonenko, 2014; Heffern et al., 2014; Wineman-Fisher et al., 2016). This binding can modify the aggregation pattern of Aβ, disrupt normal metalloenzyme activity, and produce toxic reactive oxygen species (ROS) (Bousejra-ElGarah et al., 2011; Lakatos et al., 2012; Pithadia et al., 2012; Hane and Leonenko, 2014; Heffern et al., 2014; Leong et al., 2014; Ward et al., 2015).

**Chart 1:**
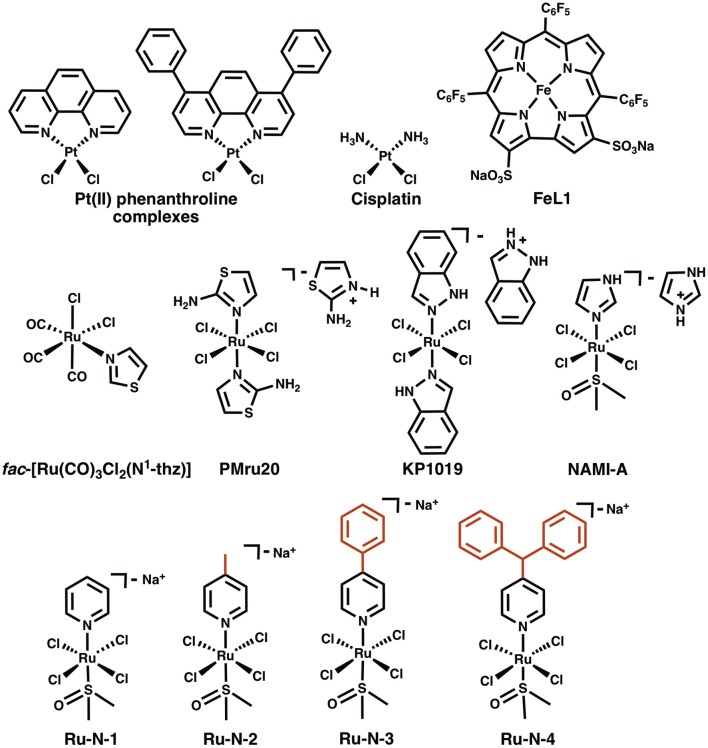
Structures of Pt(II) phenanthroline complexes, cisplatin, a Fe(III) corrole complex (FeL1), *fac*-[Ru(CO)_3_Cl_2_(N^1^-thz)], and Ru(III) complexes PMru20, KP1019, NAMI-A, and Ru(III) complexes derived from NAMI-A (**Ru-N** series).

A number of Pt (Barnham et al., 2008; Sasaki et al., 2012; Collin et al., 2013; Streltsov et al., 2013), Ru (Valensin et al., 2010; Messori et al., 2013; Jones et al., 2015), Co (Suh et al., 2007; Heffern et al., 2014; Derrick et al., 2017), and V (He et al., 2015) metal complexes have shown promise in interacting with the Aβ peptide and modifying its aggregation. For example, a series of Pt(II) phenanthroline complexes (Chart 1) were shown to bind to the peptide, modulating the aggregation and the neurotoxicity of Aβ (Barnham et al., 2008). The phenanthroline ligands were determined to facilitate π-π stacking interactions with Phe^4^, Tyr^10^, and Phe^19^ residues present in the hydrophobic region of the peptide, thus positioning the Pt(II) center in proximity to His residues (His^6,13,and14^) for covalent bond formation (Yao et al., 2004). In comparison, cisplatin (Chart 1) without large hydrophobic ligands, was shown to interact with Met^35^ (Barnham et al., 2008). For the Pt(II) phenanthroline complexes the modulation of aggregation was associated with almost complete rescuing of cell viability in primary cortical neurons, while cisplatin was inactive, demonstrating that the presence of phenanthroline ligands was essential for limiting Aβ toxicity. Barnham et al. have suggested that by coordinating to His residue(s) the Pt phenanthroline complexes inhibit the binding of ROS-generating metal ions to Aβ, such as Cu(II). This was demonstrated by a decrease in the production of H_2_O_2_ by Aβ-Cu in the presence of these complexes.

Ru(III) complexes have been investigated in anticancer research based on their cytotoxicity, relatively slow ligand exchange rate (similar to Pt(II)) (Reedijk, 2008), accessible redox chemistry in physiological conditions, and the ability to tune targeting and pharmacokinetic properties via ligand design (Webb et al., 2013). Keppler et al. were the first to report the use of Ru(III) complexes with axial azole ligands as cancer therapeutics in the 1990's (Lipponer et al., 1996; Peti et al., 1999). One of the most promising agents studied by this group was KP1019 (Chart 1) (Henke et al., 2009), which was tested in a phase I clinical trial (Hartinger et al., 2008). Recently, an analog of this compound, NKP-1339, with a Na^+^ counterion to improve water solubility, has been the focus of further development, and has also completed a phase I clinical trial (Thompson et al., 2012; Trondl et al., 2014). A second type of structurally similar Ru(III) complexes were also developed during the same time period by Alessio and co-workers (Mestroni et al., 1994; Alessio et al., 2004). These compounds have an exchangeable DMSO ligand in place of one of the axial azoles of the Keppler-type complexes. Of these, the imidazole complex NAMI-A (Chart 1) has been the most studied. This compound demonstrates less cytotoxicity than KP1019, but displays a significant antimetastatic effect, thus NAMI-A -type complexes have also been a focus for development as anticancer agents (Bergamo and Sava, 2007; Webb et al., 2012; Alessio, 2017). NAMI-A was the first Ru(III) anticancer drug to be studied in humans (Alessio, 2017), and successfully completed a phase I clinical trial, although a phase II trial demonstrated that it is only moderately tolerated according to common toxicity criteria (CTC) (Leijen et al., 2015).

The concept of Ru complexes as AD treatment agents was introduced by Valensin et al. with the report of the interaction of *fac*-[Ru(CO)_3_Cl_2_(N^1^-thz)] (Chart 1) with Aβ (Valensin et al., 2010), showing that the complex loses N^1^-thz and both Cl^−^ ligands and the ${\text{Ru}(\text{CO})}_{3}^{2+}$ unit binds to a His of the peptide. The anticancer agents PMru20 and KP1019 were also studied as a potential AD therapeutics (Messori et al., 2013). PMru20 protected rat cortical neurons from toxicity associated with both Aβ_1−42_ and the truncated Aβ_25−35_ (without His), likely by limiting peptide aggregation. KP1019 was shown to bind covalently to Aβ by modulating the peptide aggregation pattern of monomeric or pre-formed aggregates and forming soluble high-MW aggregates (Jones et al., 2015). KP1019 also limited Aβ toxicity in SH-SY5Y neuroblastoma cells.

A series of Ru(III) pyridine NAMI-A analogs (**Ru-N**, Chart 1) was reported by Walsby et al. to bind to human serum albumin (HSA), to which the use of suitable axial ligands enables tuning of the non-covalent interaction between the complexes and HSA (Webb et al., 2012). The **Ru-N** derivatives exhibited enhanced hydrophobic interactions with HSA when larger, more hydrophobic, axial pyridine-based ligands were incorporated into the NAMI-A type structure. As expected for these types of compounds, their axial DMSO ligand underwent rapid aqueous exchange at physiological pH, with loss of Cl^−^ ligands also observed. These ligand exchange processes also promoted the formation of covalent interactions with HSA, likely to His residues. Based on these observations and the previous studies described above, we hypothesized that alteration of the axial ligand in the **Ru-N** series would influence the interaction of these complexes with the Aβ peptide, with more effective peptide binding for the larger, more hydrophobic derivatives. The interaction of these Ru(III) complexes with the Aβ peptide and the associated effect on peptide aggregation are described herein.

## Materials and Methods

All common chemicals were purchased from Aldrich and used without further purification. All Ru complexes, **Ru-N-1**, **Ru-N-2**, **Ru-N-3**, and **Ru-N-4** were synthesized as reported (Webb et al., 2012). The Aβ_1−16_, and Aβ_1−42_ peptides were purchased from 21st Century Biochemicals (Marlborough, MA, USA), and Cellmano Biotech Limited (Hefei, China), and monomerized before use according to a reported procedure (Sabate et al., 2003; Pachahara et al., 2012). Aβ_1−16_ was dissolved in double distilled H_2_O (ddH_2_O), while Aβ_1−42_ was dissolved in DMSO and ddH_2_O in a 1:1 mixture, unless stated otherwise. The stock peptide solution concentration was determined by absorbance with the use of a Thermo Nicolet UV nanodrop and an extinction coefficient of 1,410 and 1,450 M^−1^cm^−1^ at 280 nm for Aβ_1−16_, and Aβ_1−42_ respectively (Guilloreau et al., 2007; Coalier et al., 2013). Turbidity assays were measured using a Synergy 4 Multi-Detection microplate reader from BioTek. ^1^H NMR spectra were recorded on a Bruker AV-600 instrument. TEM images were obtained using an OSIRIS FEI scanning TEM (STEM) operating at 200 kV.

### ^1^H NMR Binding Assay of Aβ_1−16_ Peptide to NAMI-A Derivatives

Deuterated phosphate buffered saline (PBS) (0.01 M Na_2_HPO_3_, 0.001 M KH_2_PO_4_, 0.14 M NaCl, 0.003 M KCl, pH 7.4) was prepared by removal of water by vacuum drying of PBS and dissolving the powder in D_2_O. Aβ_1−16_ was dissolved in deuterated PBS (0.01 M, pH 7.4), and **Ru-N-1** and **Ru-N-4** complexes were dissolved in DMSO-*d*_6_ and added to Aβ_1−16_ at 0.25 and 1 eq. at 10% of DMSO and the ^1^H NMR spectra were collected after approximately 15 min of incubation.

### Mass Spectrometry of Binding of Aβ_1−16_ Peptide to NAMI-A Derivatives

ESI-TOF-MS experiments were performed on an Agilent 6130 mass spectrometer connected to an Agilent 1260 HPLC system. Samples were analyzed by direct infusion (1-4 μL) of analyte into a mobile phase of 1:1 water:acetonitrile containing 5 mM ammonium acetate (pH unmodified), flowing at 0.3 mL/min and maintained at 30°C. All components of the mobile phase were mass spectrometry grade. Nitrogen drying gas was heated to 250°C, and run at 5 L/min with a nebulizing pressure of 15 psig. Voltages were: capillary 3 kV, fragmentor 175 V, skimmer 30 V, octopole 250 V. Samples were prepared as ~4 mg/mL of total protein (Aβ_1−16_) in ammonium carbonate (0.02 M, pH 9) buffer with 0 or 1 equivalents of the **Ru-N** complexes.

### Gel Electrophoresis and Western Blotting

Aβ solutions with a concentration of 25 μM were prepared in PBS (0.01 M, pH 7.4) then incubated at 37°C with continuous agitation at 200 rpm to form aggregates in the presence of **Ru-N** complexes or pyridine ligands at 1 eq. Samples were collected at 3, 6, 11, and 24 hour time points. Concentration-dependent modulation of Aβ aggregation was also evaluated after 24 hour incubation for **Ru-N-1** and **Ru-N-4** (0.25, 0.5, 1, and 2 equivalents). Electrophoresis separation of peptide aggregates was completed using 8–16% Mini-PROTEAN® TGX Precast Gels from Bio-Rad, at 100 V for 100 min. The gels were then transferred to a nitrocellulose membrane for 1 hour at 100 V at 4°C, followed by blocking of the membrane in a 3% BSA solution in Tris-buffered saline (TBS) (0.02 M Tris, 0.15 M NaCl, 0.003 M KCl) for 1 h. The membrane was incubated in a solution (1:2,000 dilution) of 6E10 anti-Aβ primary antibody (Biolegends) overnight. After washing 5 × 5 min with TBS, the membrane was incubated in a solution containing the secondary antibody (Horseradish peroxidase, Caymen Chemicals) for 3 h. A Thermo Scientific SuperSignal® West Pico Chemiluminescent Substrate kit was used to visualize the Aβ species using a BioRad ChemiDoc^TM^ MP imaging system.

### Transmission Electron Microscopy (TEM)

Samples were prepared from the Western blot assay after the 24 h incubation time at 37°C. TEM grids were prepared following previously reported methods (Jones et al., 2012). In order to increase hydrophilicity of the Formvar/Carbon 300-mesh grids (Electron Microscopy Sciences), the grids were glow discharged in a vacuum for 10 s. Drops of samples (10 μL) were placed onto a sheet of parafilm and the TEM grid was laid on the drop for 5 min. The grid was then placed on a drop of syringe-filtered 5% uranyl acetate and then immediately removed. This process was then repeated for a second drop of 5% uranyl acetate. Finally, the grid was placed on a third drop of 5% uranyl acetate and incubated for 1 min. Excess uranyl acetate was removed using a tissue between drops. The grid was allowed to air-dry for at least 15 min. Bright-field images were obtained on a FEI Tecnai Osiris STEM at 200 kV.

### Turbidity Assay

The turbidity assay was conducted in quadruplicate in flat-bottomed 96-well assay plates (Microtest, BD Falcon). Aβ_1−42_ peptide and **Ru-N** complexes had final concentrations of 10 μM. **Ru-N** complexes were dissolved in DMSO and further diluted to obtain the correct concentration. The absorbance at 500 nm was measured every 10 min for 3 h at 37°C under constant agitation using a Synergy 4 Fluorometer plate reader from BioTek. For the 20 h experiment, the samples were incubated at 37°C with constant agitation with a lid on to prevent evaporation and then the turbidity was measured.

### Bradford Assay

The Bradford assay (Thermo Scientific) measures the absorbance at 595 nm of Coomassie brilliant blue G-250 as it binds to protein in duplicate in a flat-bottomed 96-well assay plate (Microtest, BD Falcon). Sixty microliter solutions of Aβ_1−42_ in the presence of 1 eq. of **Ru-N** complexes were incubated at 37°C for 24 h under constant agitation. A 30 μL sample was removed at the beginning of the experiment as the 0 hour time point, and kept frozen at −80°C until time for absorbance reading. Samples were centrifuged prior to reading of the assay to remove insoluble fibrils (Mok and Howlett, 2006). Measurements of absorbance used a Synergy 4 Fluorometer plate reader from BioTek. Samples were measured in duplicate, and statistics completed using the PRYSM program and ANOVA.

## Results

### Binding of Aβ His Residues to Ru-N Derivatives

**Figure 1:**
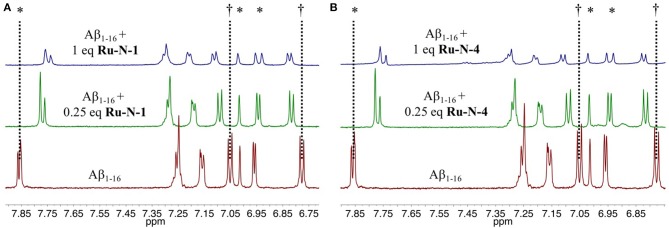
Changes in the ^1^H NMR spectra of Aβ_1−16_ in the presence of **Ru-N** derivatives. Shown are spectra obtained from 205 μM Aβ_1−16_, in pH 7.4 PBS/D_2_O buffer at 25°C (red) with addition of 0.25 eq. (green) and 1 eq (blue) of **(A) Ru-N-1** or **(B) Ru-N-4**. *His^6^, His^13^ and His^14^. ^†^Tyr^10^.

To evaluate the nature of the interactions between the **Ru-N** complexes and the Aβ peptide, ^1^H NMR of Aβ_1−16_ in the presence of paramagnetic (Ru(III), *d*^5^, *S* = ½) **Ru-N-1** or **Ru-N-4** were obtained at 0.25 and 1 equivalents (Figure 1). These complexes were selected as they exhibit the largest difference in pyridine ligand size. In addition, the non-aggregating Aβ_1−16_ peptide fragment was used, which includes the metal binding amino acid residues. Upon addition of either Ru(III) complex, all signals for the residues in Aβ_1−16_ exhibit a shift, suggesting that an interaction between peptide and complex is occurring. Interestingly there is a significant decrease in the intensity as well as a broadening of the signals of Aβ_1−16_ in the presence of 1 eq of the paramagnetic complexes. We do not observe a precipitate in the NMR tube in our experiments. The largest shift (ca. 0.1 ppm) observed is for the His resonance at 7.85 ppm, which suggests binding of a peptide His residue. This mode of coordination has also been reported for interaction of these complexes with HSA (Webb et al., 2012).

**Figure 2:**
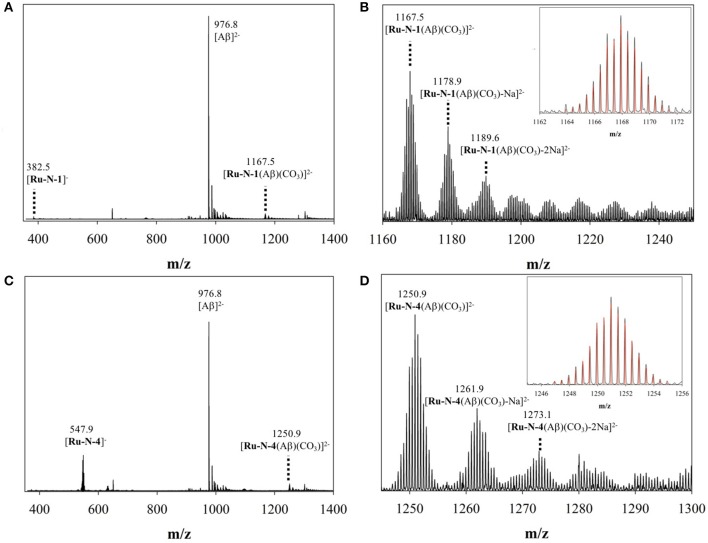
ESI-MS of **(A)** binding of Aβ_1−16_ to **Ru-N-1**; **(B)** zoomed region of [**Ru-N-1**(Aβ)(CO_3_)]^2−^ with associated Na adducts; and **(C)** binding of Aβ_1−16_ to **Ru-N-4**; **(D)** zoomed region of [**Ru-N-4**(Aβ)(CO_3_)]^2−^ with associated Na adducts. Across all experiments, species were observed as [**Ru-N**_(0−1)_(Aβ)(CO_3_)Na_(0−6)_]^2−^.

To investigate further the interaction between the complexes and the peptide, ESI mass spectrometry was performed on solutions of Aβ_1−16_ incubated with either **Ru-N-1** or **Ru-N-4**. The mass spectra (Figure S1, Figures 2A,C, respectively) indicate the formation of the adducts [**Ru-N-1**(Aβ_1−16_)(CO_3_)]^2−^ (m/z = 1167.5) and [**Ru-N-4**(Aβ_1−16_)(CO_3_)]^2−^ (m/z = 1250.9), where carbonate (${\text{CO}}_{3}^{2-}$) in the adducts is likely derived from the running buffer ([NH_4_]^2+^ [CO_3_]^2−^) used in the MS experiment. The characteristic Ru isotopic pattern was observed for both peaks (Figures 2B,D), and the masses of the adducts are consistent with loss of the DMSO ligand from each Ru complex, and subsequent coordination to the Aβ_1−16_ peptide.

### Influence of Ru-N Series on Aβ Aggregation

**Figure 3:**
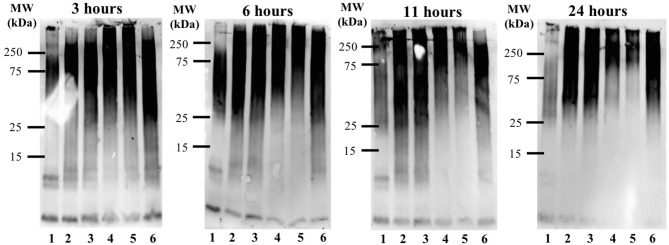
Influence of **Ru-N** derivatives on the aggregation profile of Aβ_1−42_. Gel electrophoresis/Western blot of 25 μM Aβ_1−42_ and 1 eq. of **Ru-N** derivatives in PBS buffer (0.01 M, pH 7.4) at incubation timepoints 3, 6, 11, and 24 h, with constant agitation at 37°C, using anti-Aβ antibody 6E10. Lane 1: Aβ_1−42_; lane 2: Aβ_1−42_ + **Ru-N-1**; lane 3: Aβ_1−42_ + **Ru-N-2**; lane 4: Aβ_1−42_ + **Ru-N-3**; lane 5: Aβ_1−42_ + **Ru-N-4**; lane 6: Aβ_1−42_ + **NAMI-A**.

The time-dependent influence of the **Ru-N** derivatives on Aβ_1−42_ aggregation was analyzed via gel electrophoresis and western blotting, in combination with Transmission Electron Microscopy (TEM). The Aβ_1−42_ peptide was chosen for these experiments due to its high propensity for aggregation and significant neurotoxicity (Gong et al., 2003; Haass and Selkoe, 2007; Walsh and Selkoe, 2007; Jakob-Roetne and Jacobsen, 2009; Kepp, 2012; Nortley et al., 2019; Zott et al., 2019). At each time point (3, 6, 11, and 24 h) a 30 μL aliquot was removed from the stock incubation solution for each treatment and kept at −80°C until further analysis. An increase in high MW aggregates over time was observed for Aβ_1−42_ alone (Lane 1, Figure 3), with a significant decrease in soluble Aβ species at 24 h, as expected based on prior results (Jones et al., 2015; Gomes et al., 2019). The **Ru-N** derivatives do not significantly affect aggregation at the 3 h timepoint. However, at longer timepoints the complexes generate increased soluble higher molecular weight species in comparison to peptide alone. This effect is most pronounced for the complexes **Ru-N-3** (lane 4) and **Ru-N-4** (lane 5), which have the largest pyridine-derived ligands. The Ru(III) complexes containing smaller pyridine-derived ligands, such as **Ru-N-1** (lane 2) and **Ru-N-2** (lane 3), show a similar modulation of Aβ aggregation to **NAMI-A** (lane 6), which could reflect the similar properties of the pyridine, 6-methyl-pyridine, and imidazole ligands in this assay. Interestingly, the Na[Ru(DMSO)_2_Cl_4_] complex without an apical aza ligand also induces the formation of soluble high molecular weight Aβ species after 24 h incubation (Figure S2), however the molecular weight range is larger (~25–250 kDa), even in comparison to **Ru-N-1**. Overall, these results indicate that in comparison to the formation of insoluble peptide aggregates for peptide alone at 24 h, 1 eq. of the **Ru-N** complexes promotes the formation of soluble high molecular weight aggregates.

In order to determine if the pyridine ligands alone can influence Aβ aggregation, Aβ_1−42_ aggregation was evaluated at 3, 6, 11, and 24 h by gel electrophoresis and western blotting in the presence of 1 eq. of the free pyridine ligands. As expected, a decrease in monomeric species and an increase in high MW species is observed for peptide alone over the incubation period (Figure S3). Interestingly, the presence of 1 eq. of the pyridine ligands does not significantly change the Aβ_1−42_ aggregation pattern (Figure S3), indicating that the Ru(III) complex, and not the pyridine ligand, is essential for influencing Aβ peptide aggregation.

**Figure 4:**
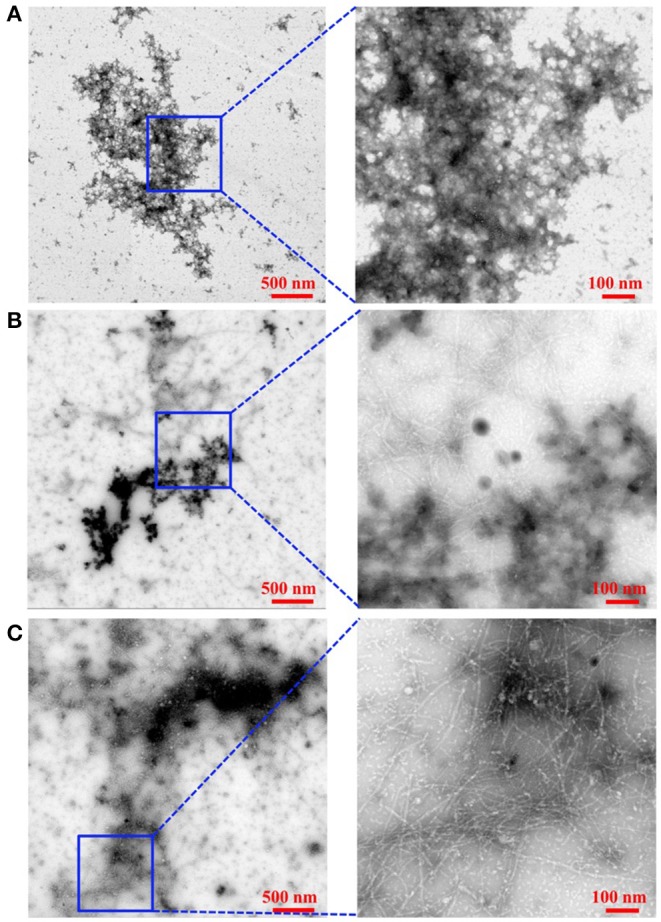
Influence of **Ru-N-1** and **Ru-N-4** on the aggregation profile of Aβ_1−42._ TEM of Aβ_1−42_ alone **(A)**, Aβ_1−42_ with 1 eq. of **Ru-N-1 (B)**, and Aβ_1−42_ with 1 eq. of **Ru-N-4 (C)** incubated for 24 h with agitation at 37°C. Box shows where higher magnification image was obtained.

**Figure 5:**
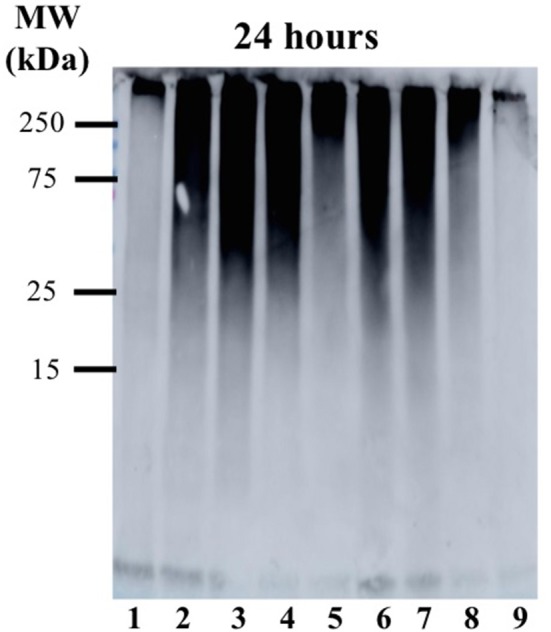
Gel electrophoresis/Western blot of 25 μM Aβ_1−42_ and different concentrations of **Ru-N-1** and **Ru-N-4** in PBS buffer (0.01 M, pH 7.4) at 24 h incubation with agitation at 37°C, using anti-Aβ antibody 6E10. Lane 1: Aβ_1−42_; lane 2: Aβ_1−42_ + 0.25 eq. **Ru-N-1**; lane 3: Aβ_1−42_ + 0.5 eq. **Ru-N-1**; lane 4: Aβ_1−42_ + 1 eq. **Ru-N-1**; lane 5: Aβ_1−42_ + 2 eq. **Ru-N-1**; lane 6: Aβ_1−42_ + 0.25 eq. **Ru-N-4**; lane 7: Aβ_1−42_ + 0.5 eq. **Ru-N-4**; lane 8: Aβ_1−42_ + 1 eq. **Ru-N-4**; lane 9: Aβ_1−42_ + 2 eq. **Ru-N-4**.

TEM images (Figure 4 and Figure S5) of Aβ_1−42_ alone and in the presence of either **Ru-N-1** or **Ru-N-4** after incubation for 24 h show different morphologies for the three samples analyzed. Aβ_1−42_ incubated alone led to the formation of large amorphous aggregates, with no fibrils observed on the TEM grid. The presence of the **Ru-N** complexes led to an increase in fibril formation, with both **Ru-N-1** and **Ru-N-4** showing a mixture of fibrils and amorphous aggregates. The size of the amorphous aggregates are however much larger for peptide alone in comparison to treatment with the **Ru-N** derivatives (Figure S5). These results are consistent with the electrophoresis gels (Figure 3) that show that most of the peptide has formed large insoluble aggregates for peptide alone, while the **Ru-N** series show stabilization of smaller soluble aggregates. The concentration-dependent effect of the Ru complexes (**Ru-N-1** and **Ru-N-4**) on Aβ_1−42_ aggregation was also investigated at the 24-h timepoint. In this case, the complexes **Ru-N-1** and **Ru-N-4** were added to Aβ_1−42_ at 0.25, 0.5, 1, and 2 eq. and the aggregation pattern investigated by gel electrophoresis and western blotting (Figure 5). Lane 1 shows high MW aggregates for peptide alone after 24 h of incubation. The presence of **Ru-N-1** has a concentration-dependent effect on aggregation, with aggregates of 25 kDa and higher for 0.25, 0.5, and 1 eq., whereas 2 eq. leads to formation of aggregates of *ca*. 150 kDa and higher. Interestingly, **Ru-N-4** shows a more pronounced concentration-dependent change in Aβ aggregation, with incubation of 1 eq. of **Ru-N-4** resulting in aggregates of *ca*. 150 kDa or higher and 2 eq. affording aggregates higher than *ca*. 250 kDa in MW. These results indicate a greater shift to high MW aggregates for **Ru-N-4** (incorporating the bulky pyridine-derived ligand) than for **Ru-N-1**.

**Figure 6:**
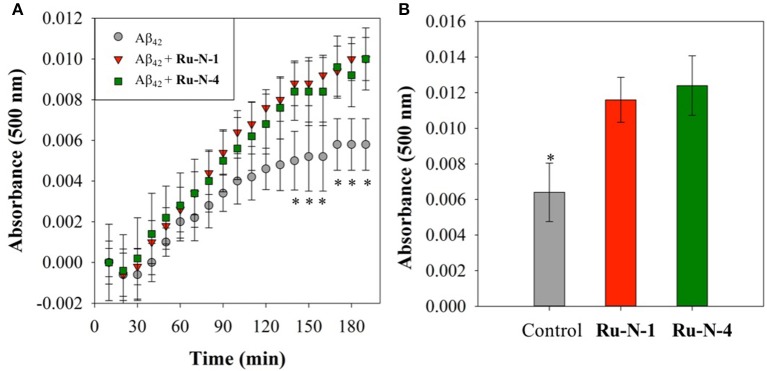
Turbidity assay data from 10 μM Aβ_1−42_ (gray) and with 1 eq. of **Ru-N-1** (red) or **Ru-N-4** (green) in PBS buffer (0.01 M, pH 7.4) with agitation at 37°C. **(A)** Change in absorbance during initial 3 h of incubation. **(B)** Change in absorbance at 20 h of incubation. *Statistically significant difference between Aβ_1−42_ only and in the presence of the complexes, *p* < 0.05 for **(A)** and *p* = 0.01 for **(B)**. Calculated using JMP13 (Wilcoxon method).

**Figure 7:**
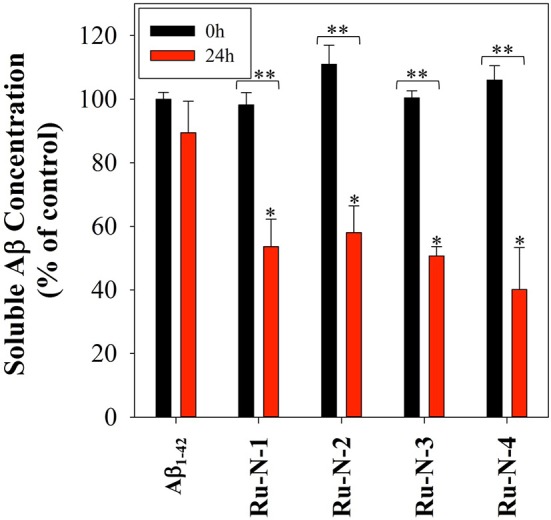
Bradford assay of 60 μM Aβ_1−42_ in the presence of 1 eq. of all four **Ru-N** complexes in PBS buffer (0.01 M, pH 7.4) at 0 hour (black) and after 24 h of incubation with agitation at 37°C (red). Samples were centrifuged at 14,000 g for 5 min prior to absorbance measurement. Statistically significant difference between *Aβ_1−42_ only and in the presence of the complexes (**Ru-N-2**, *p* = 0.0025, **Ru-N-1** and **Ru-N-3**, *p* = 0.0005, and **Ru-N-4**, *p* < 0.0001) **0 and 24 h time point (**Ru-N-1**, *p* = 0.0003, and **Ru-N-2**, **Ru-N-3**, and **Ru-N-4**, *p* < 0.0001). Calculated using 2 way ANOVA.

The Aβ aggregation process in solution can be studied by a number of different methods, including turbidity (Storr et al., 2007; Gomes et al., 2014; Barykin et al., 2018), dynamic light scattering (Davis et al., 2009; Nichols et al., 2015), and Thioflavin T (ThT) fluorescence (Barnham et al., 2008; Jones et al., 2015). We have previously shown that the Ru(III) complex KP1019 interferes with ThT fluorescence analysis (either by quenching or inhibition of ThT binding) (Jones et al., 2015), however, turbidity has been shown to be a reliable alternative for the investigation of peptide aggregation in the presence of compounds that disrupt ThT fluorescence (Cook and Martí, 2012). We thus employed turbidity measurements here to investigate the effect of **Ru-N-1** and **Ru-N-4** on Aβ_1−42_ aggregation in solution. The formation of peptide aggregates in solution over time leads to an increase in turbidity, and the degree of light scattering can be measured by UV-vis measurements (Gomes et al., 2014). The results of the time-dependent aggregation of Aβ_1−42_ in the presence of **Ru-N** by electrophoresis and western blot (Figure 3) show that the complexes appear to induce the formation of soluble higher MW aggregates after 24 h of incubation. At this time point the aggregation profiles for **Ru-N-1** and **Ru-N-4** differ indicating an effect of the axial ligands. In order to further evaluate the influence of the **Ru-N** series on peptide aggregation in solution, the turbidity of an Aβ_1−42_ solution was measured in quintuplicate in a 96-well plate over the course of 3 h in the presence and absence of **Ru-N-1** and **Ru-N-4** (Figure 6A). Aggregation was monitored at 500 nm as there is no absorption by either the Ru complexes or the peptide at this wavelength. As expected, an increase in turbidity was observed for the peptide alone over the 3-h incubation period. In the presence of the Ru(III) complexes an increase in turbidity was also observed, and at the 2 h timepoint the presence of **Ru-N-1** and **Ru-N-4** results in a significant increase in turbidity in comparison to peptide alone, but with no statistical difference between the two complexes. Due to water evaporation from the 96-well plate at longer measurement times, a lid was placed on the plate at 3 h, and a further single reading taken at the 20 h timepoint (Figure 6B). At the longer timepoint an approximate doubling of the turbidity is observed for solutions containing the Aβ_1−42_ peptide and either **Ru-N-1** and **Ru-N-4** complexes in comparison to peptide alone. Again, no statistical difference between the two complexes was observed (Chart 1). Overall, the higher turbidity reading for the **Ru-N** complexes in comparison to peptide alone is likely due to the formation of a large number of soluble aggregates for the former, while fewer insoluble peptide aggregates form for the latter. This conclusion is in accord with the gel studies, and TEM data. We next investigated the total amount of Aβ_1−42_ peptide after incubation with and without the **Ru-N** complexes via the Bradford assay. The Bradford assay measures the shift in the absorbance peak for the reagent Coomassie brilliant blue G-250 from 495 nm to 595 nm upon binding to the C-terminus of proteins (Bradford, 1976). Before measurement the samples were centrifuged to remove insoluble fibrils using an established protocol (Wang et al., 2002; Mok and Howlett, 2006). We analyzed the change in the concentration of Aβ_1−42_ between 0 and 24 h of incubation in the presence of the four **Ru-N** complexes. The peptide alone does not show a significant decrease in the amount of peptide at 24 h of incubation (Figure 7), suggesting that the aggregates at this stage are non-fibrillar. This result is in accord with TEM images showing only amorphous aggregates for peptide alone (Figure 4). In contrast, after 24 h of incubation in the presence of the **Ru-N** complexes, a decrease in the amount of peptide is observed, suggesting that fibrillar species had formed and were removed via centrifugation. This result is also in accord with fibril formation observed for **Ru-N** treatments by TEM. Overall, the complexes reduce the amount of measurable Aβ in the sample by 50% after 24 h, with no significant difference observed across the **Ru-N** series.

## Discussion

The modulation of Aβ peptide aggregation in the presence of the **Ru-N** series of complexes, as well as the ability of these complexes to bind to the peptide, have been described in this study. The **Ru-N** series contains four NAMI-A derivatives with pyridyl ligands of different sizes (Chart 1), and it was hypothesized that an enhanced interaction with the Aβ peptide would occur for the larger more hydrophobic derivatives. Both non-covalent and covalent interactions of these complexes with HSA have been characterized by Walsby et al., using electron paramagnetic resonance (EPR) (Webb et al., 2012). HSA has 16 His residues, of which 5 His residues are available at the surface of the protein (Hnizda et al., 2008), providing binding sites for metal ions. The major species formed upon incubation of HSA with the **Ru-N** series are His adducts at both the axial and equatorial positions following the loss of DMSO or Cl ligands (Webb et al., 2012). Interestingly, the interaction of NAMI-A with a number of proteins including lysozyme (Messori and Merlino, 2014), carbonic anhydrase (Casini et al., 2010), and human H-chain ferritin (Ciambellotti et al., 2018) has been studied by X-ray crystallography (Alessio and Messori, 2019). In these studies all of the original ligands of NAMI-A are released, and the resulting Ru(III) center is bound to the protein via His, Asp, and Glu side-chains. However, it has been postulated that the process of crystal soaking, in which NAMI-A crystallizes with the protein, can lead to different binding/speciation in comparison to solution studies (Alessio, 2017). **Ru-N-1** (also called AziRu) has also been reported to exchange all ligands when binding to lysozyme (Vergara et al., 2013a), and RNase A (Vergara et al., 2013b). The binding site for lysozyme involves His^15^, Arg^14^, and Asp^87^, while **Ru-N-1** binds to RNase A via a single His. Interestingly, even though RNase A contains four solvent-exposed His, only one Ru-His adduct is formed per RNase molecule, to which water molecules complete the distorted octahedral coordination sphere.

The results of the ESI-MS studies herein are consistent with the prior work with HSA (Webb et al., 2012), showing adduct formation for both **Ru-N-1** and **Ru-N-4** with Aβ_1−16_ via loss of an exchangeable DMSO ligand. In addition, incubation of **Ru-N-1** or **Ru-N-4** with Aβ_1−16_ led to a shift and broadening of all of the ^1^H NMR signals of the Aβ peptide, suggesting an interaction between the Ru(III) complexes and Aβ_1−16_ (Figure 1). Similar line broadening of Aβ ^1^H NMR signals has been observed in the presence of Cu(II) (Eury et al., 2011) and an Fe(III) corrole complex (FeL1, Chart 1) (Gomes et al., 2019), along with the disappearance or shifting of the His resonances. This has been interpreted as binding of either Cu(II) or FeL1 to His residues present in the hydrophilic portion of the peptide. In another report (Valensin et al., 2010), broadening of the ^1^H NMR spectrum of Aβ_1−28_, and the almost complete disappearance of the aromatic signals for His and Tyr, was observed upon incubation of *fac*-[Ru(CO)_3_Cl_2_(N^1^-thz)] (Chart 1) with the peptide. These results supported Aβ_1−28_ His binding to the Ru(II) complex with ESI-MS verification of adduct formation (Valensin et al., 2010). Although all the peptide NMR signals shift upon interaction with the **Ru-N** complexes in this work, the peptide His resonance at 7.85 ppm undergoes the largest change (*ca*. 0.1 ppm), which is consistent with what has been observed for metal ions or complexes with Aβ (Eury et al., 2011; Gomes et al., 2019). Interestingly, weak signals attributed to the free pyridine ligand at 7.35 ppm and 7.45 ppm are observed upon addition of 1 eq. **Ru-N-4** to Aβ_1−16_ (Figure 1), and these signals increase in intensity at 24 h for 0.25 eq of **Ru-N-4** (Figure S4). Pyridine ligand loss is not observed for the **Ru-N-1** complex (Figure 1 and Figure S3), suggesting that pyridine ligand exchange is enhanced for the more bulky hydrophobic **Ru-N-4** complex. The presence of the free pyridine ligand of **Ru-N-4** upon incubation with Aβ_1−16_ suggests further ligand exchange processes occur for this derivative in addition to DMSO exchange, similar to the reported X-ray studies (Casini et al., 2010; Messori and Merlino, 2014; Ciambellotti et al., 2018), and this difference between **Ru-N-1** and **Ru-N-4** may play a role in the peptide aggregation process (*vide infra*).

Several metal complexes have been reported to modulate the aggregation pattern of Aβ upon binding covalently to the peptide (Collin et al., 2013; Kenche et al., 2013; Heffern et al., 2014; Jones et al., 2015; Gomes et al., 2019). For example, the binding of the Fe(III) corrole complex FeL1 (Chart 1) to Aβ lead to the stabilization of low MW oligomeric species (Gomes et al., 2019), however, binding of KP1019 (Chart 1) led to decreased oligomer formation and an increase in high MW soluble aggregates (Jones et al., 2015). The Ru(III) complexes investigated in this study have a similar effect to that observed for KP1019, leading to the formation of soluble high MW aggregates in a concentration-dependent manner. Our results also show that the binding of the Ru(III) center is essential for the change in aggregation, since the ligands alone do not exhibit an effect on the aggregation process. The electrophoresis gel/western blot data suggests a greater influence on aggregation by the **Ru-N** complexes with larger, more hydrophobic ligands (**Ru-N-3** and **Ru-N-4**). In addition, the complex without the apical Py ligand, leads to a range of soluble species after 24 h aggregation, with the gel results similar to NAMI-A. The fibrillar structures shown by TEM in the presence of **Ru-N-1** and **Ru-N-4** when compared to the amorphous aggregates for peptide alone, suggest that binding of the complexes to Aβ promotes fibrillization of the peptide.

Additionally, incubation of the **Ru-N** series with the Aβ peptide for 24 h, followed by centrifugation, leads to a 50% decrease in peptide concentration in comparison to peptide alone as determined by a Bradford assay. We employed a centrifugation protocol to remove insoluble fibrils (Wang et al., 2002; Mok and Howlett, 2006), and thus the reduction in peptide measured for the **Ru-N** treatments is likely due to the removal of fibrillar structures, as observed by TEM. Alternatively, the Bradford assay depends on Coomassie blue binding to basic amino acids (such as His), thus it is possible that **Ru-N** binding to the peptide leads to the observed reduction in signal. However, we would expect to see a reduction in signal in the initial measurements due to interaction of the **Ru-N** complexes with the peptide if this was the case.

Overall, the **Ru-N** series promote the formation of soluble high molecular weight aggregates at 24 h, while peptide alone leads to almost complete precipitation of the peptide. Only minor differences are observed across the **Ru-N** series, with the larger more hydrophobic derivatives (**Ru-N-3** and **Ru-N-4**) narrowing the size distribution of the soluble aggregates to higher molecular weights (Figure 3). TEM analysis (Figure 4) of the insoluble aggregates shows that while incubation of peptide alone produces very large amorphous aggregates, **Ru-N** treatment results in both fibrils and amorphous aggregates, with the amorphous aggregates smaller in size in comparison to peptide alone. It is possible that by stabilizing soluble high molecular weight species, the Ru-N complexes slow down the rate of peptide precipitation, thereby promoting the formation of the more ordered fibrillar structures observed by TEM. Our results suggest that increasing the pyridine ligand size/hydrophobicity even further may afford fibrillar structures exclusively, which could ultimately have a protective effect in AD by promoting the formation of a stable insoluble peptide aggregate with limited potential to furnish toxic oligomeric species (Treusch et al., 2009; Iadanza et al., 2018; Mroczko et al., 2018).

## Conclusions

This study highlights the ability of a series of Ru(III) complexes derived from NAMI-A to interact with the Aβ peptide and modify aggregation, a known hallmark of AD. It has been shown that the DMSO ligand of the **Ru-N** complexes can readily be exchanged in buffer (likely for H_2_O), which provides a binding site for His residues when incubated with proteins, such as HSA (Webb et al., 2013). Our NMR and ESI-MS results are in accord with the previous findings of binding of metal ions or complexes to Aβ and support a covalent interaction of the **Ru-N** complexes with His residues of the Aβ peptide. The effect of changing the size of the pyridine-derived ligands in the **Ru-N** series on Aβ aggregation was also investigated, and an increase in the size and hydrophobicity of the pyridine-derived ligand leads to larger-sized aggregates. The influence of **Ru-N-3** and **Ru-N-4** on peptide aggregation is demonstrated to be greater than that of the smaller complexes **Ru-N-1** and **Ru-N-2**, with a more prominent induction of soluble high MW aggregates, as demonstrated by electrophoresis gel and western blotting. A concentration-dependent modulation of aggregation was demonstrated for **Ru-N-1** and **Ru-N-4**, where addition of 2 equivalents of the first complex has a comparable effect on peptide aggregation as 1 equivalent of the latter. Interestingly, the aggregation of Aβ_1−42_ alone after 24 h shows only large amorphous aggregates by TEM, while the presence of 1 equivalent of either **Ru-N-1** or **Ru-N-4** shows formation of smaller amorphous aggregates as well as fibrils. However, investigation of the aggregation process in solution, by turbidity analysis, does not distinguish between the **Ru-N** complexes in terms of peptide aggregation. The **Ru-N-1** and **Ru-N-4** complexes exhibit increased turbidity in comparison to peptide alone at 3 and 24 h, consistent with formation of a greater number of aggregates in comparison to peptide alone. Interestingly, all four **Ru-N** complexes exhibit a ca. 50% decrease in peptide concentration in comparison to peptide alone via a Bradford assay. This result is likely due to the removal of insoluble fibrils in the **Ru-N** samples (observed by TEM) via centrifugation. In this work we have shown that the **Ru-N** series undergoes ligand exchange and covalent binding to the Aβ peptide, which leads to modulation of the peptide aggregation pathway, promoting the formation of high molecular weight aggregates in solution, with both amorphous and fibrillar aggregate morphology. Further investigation of the pharmacokinetic properties of the **Ru-N** complexes, and influence of these complexes on the toxicity of Aβ in cell assays, will provide insight into their therapeutic potential.

## Data Availability Statement

All datasets generated for this study are included in the article/Supplementary Material.

## Author Contributions

LG, JB, AJ, and JS: investigation. LG: writing—original draft preparation and visualization. TS and CW: writing—review and editing, supervision, and funding acquisition.

### Conflict of Interest

The authors declare that the research was conducted in the absence of any commercial or financial relationships that could be construed as a potential conflict of interest.
