# Supplementary material for: Modification of Aβ Peptide Aggregation via Covalent Binding of a Series of Ru(III) Complexes
Source: Front Chem. 2019 Dec 3;7:838. doi: 10.3389/fchem.2019.00838 (PMC6915085; doi:10.3389/fchem.2019.00838)
Supplement: Supplementary file 1 [file Data_Sheet_1.PDF]

Modification of Aβ Peptide Aggregation via Covalent Binding of a Series of Ru(III) Complexes

Luiza M.F. Gomes[1], Janaina C. Bataglioli[1], Allison J. Jussila[1], Jason R. Smith[1], Charles J. Walsby[1,]* and Tim Storr[1,]*

Supplementary Information

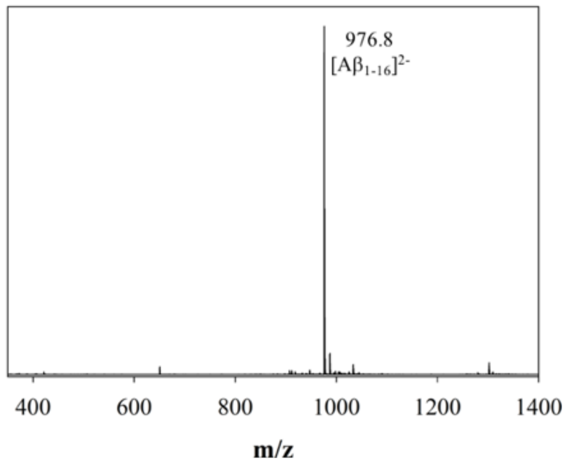

Figure S1. ESI-MS of A$\beta_{1\text{-}16}$ with its sodium adducts. Across all experiments, species were observed as $[A\beta Na_{(0\text{-}6)}]^{2-}$. The dimeric species $[A\beta_2\text{-}Cl]^{3-}$ at m/z 1303-1374.

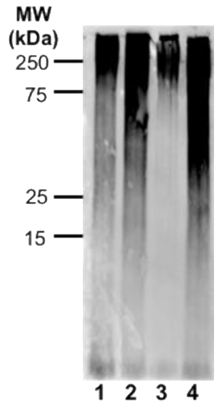

Figure S2. Influence of $[Ru(DMSO)_2Cl_4]Na$ on the aggregation profile of A$\beta_{1\text{–}42}$. Gel electrophoresis/Western blot of 25 μM A$\beta_{1\text{–}42}$ and 1 eq. of Ru(III) complexes in PBS buffer (0.01 M, pH 7.4) at 24 hour incubation, with constant agitation at 37 °C, using anti-Aβ antibody 6E10. Lane 1: A$\beta_{1\text{–}42}$; lane 2: A$\beta_{1\text{–}42}$ + **Ru-N-1**; lane 3: A$\beta_{1\text{–}42}$ + **Ru-N-4**; lane 4: A$\beta_{1\text{–}42}$ + $[Ru(DMSO)_2Cl_4]Na$. The high MW species in the peptide alone lane in comparison to Figure 3 could be due to the loading of a peptide suspension on the gel (lack of settling) and that the small percentage of SDS could have partially broken up the amorphous aggregates.

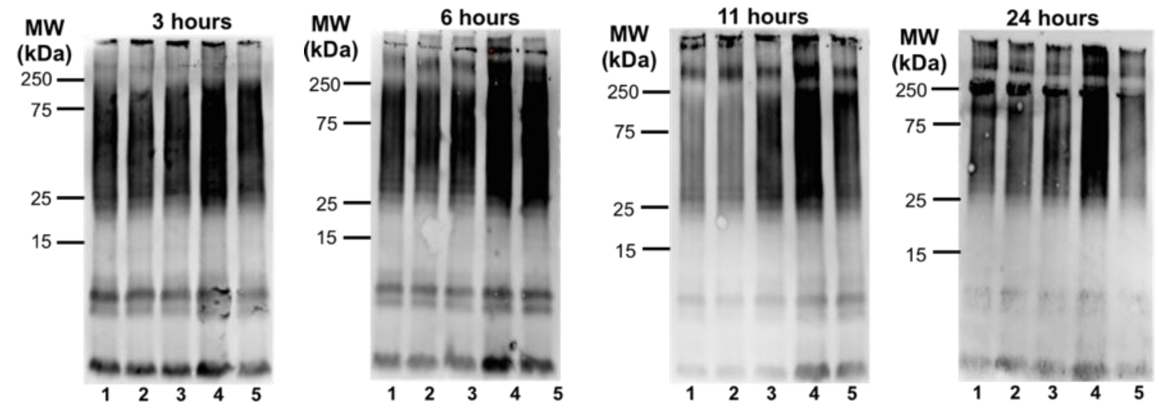

Figure S3. Influence of pyridine ligands on the aggregation profile of A$\beta_{1\text{–}42}$. Gel electrophoresis/Western blot of 25 µM A$\beta_{1\text{–}42}$ and 1 eq. of pyridine ligands in PBS buffer (0.01 M, pH 7.4) at incubation time points 3, 6, 11 and 24 hours, with constant agitation at 37 °C, using anti-Aβ antibody 6E10. Lane 1: A$\beta_{1\text{–}42}$; lane 2: A$\beta_{1\text{–}42}$ + **L1**; lane 3: A$\beta_{1\text{–}42}$ + **L2**; lane 4: A$\beta_{1\text{–}42}$ + **L3**; lane 5: A$\beta_{1\text{–}42}$ + **L4**.

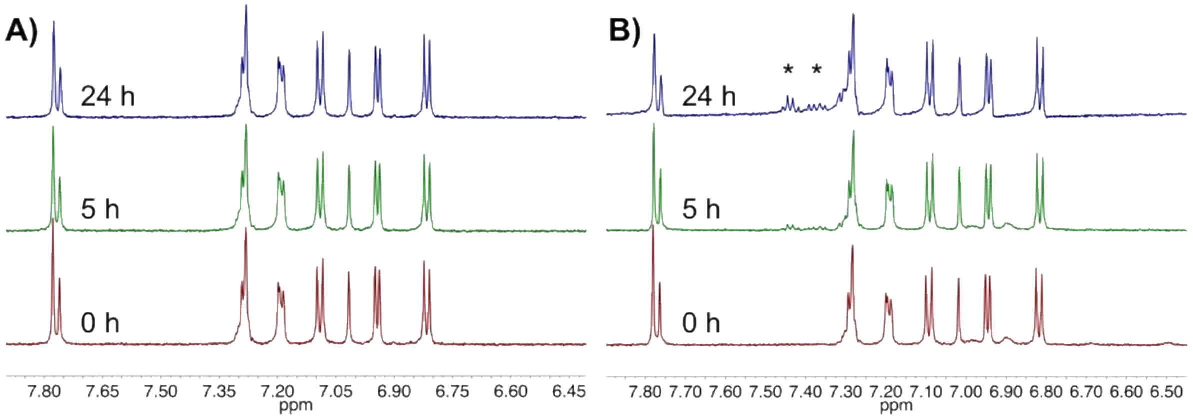

Figure S4. Changes in the ^{1}H NMR spectra of A$\beta_{1\text{-}16}$ in the presence of 0.25 eq. of **Ru-N** derivatives incubated over time with constant agitation at 37 °C. Shown are spectra obtained from 205 µM A$\beta_{1\text{-}16}$, in pH 7.4 PBS/D_2O buffer at 0 h (red) 5 h (green) and 24 h (blue) of **A) Ru-N-1** or **B) Ru-N-4**. ***free ligand**.

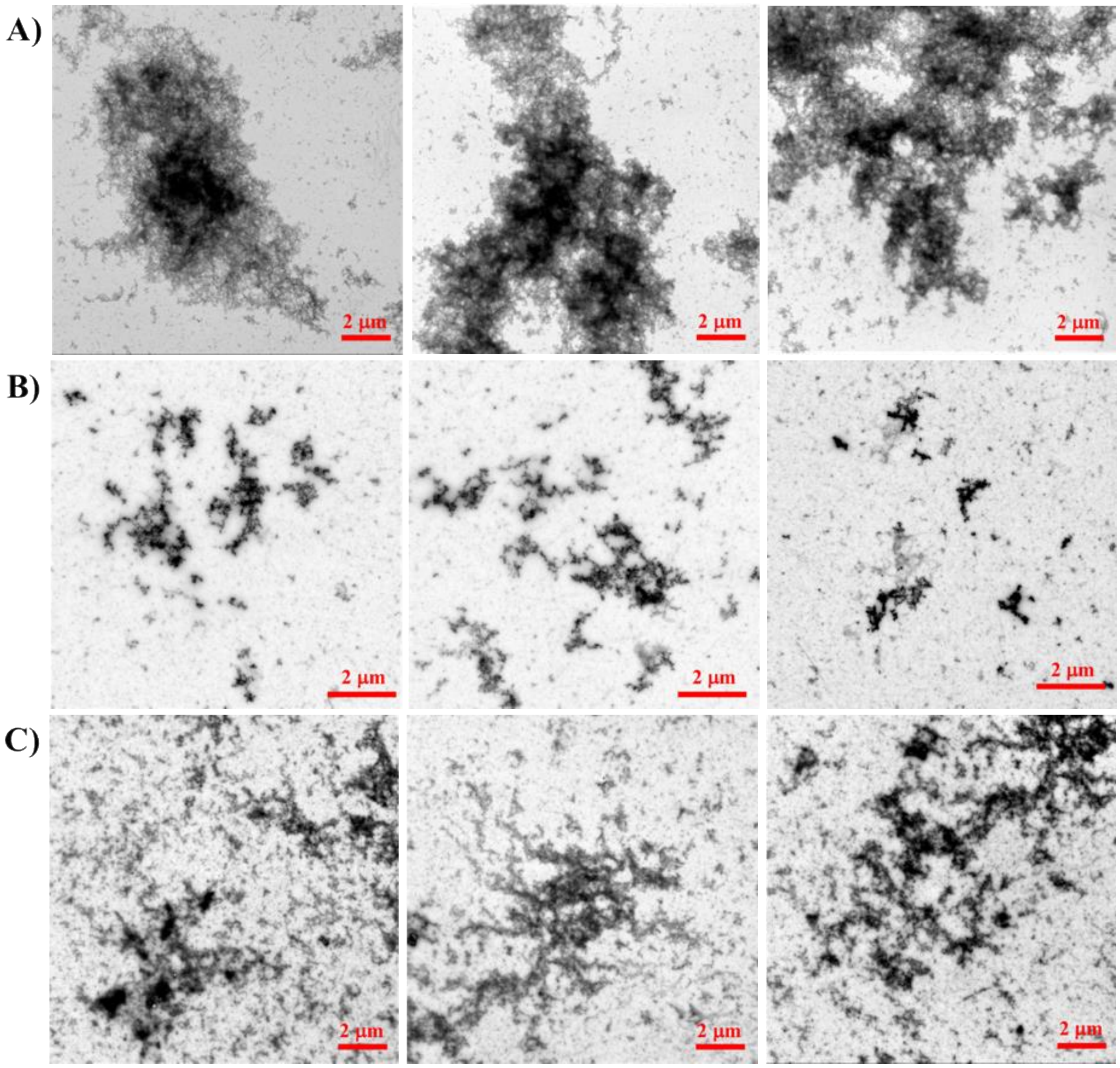

Figure S5. Influence of **Ru-N-1** and **Ru-N-4** on the aggregation profile of Aβ_{1-42}. TEM of Aβ_{1-42} alone (A), Aβ_{1-42} with 1 eq. of Ru-N-1 (B) and Aβ_{1-42} with 1 eq. of Ru-N-4 (C) incubated for 24 hours with agitation at 37 ˚C.
